# Chronische Rückenschmerzen bei axialer Spondyloarthritis: Aktuelle diagnostische Herausforderungen und therapeutische Möglichkeiten

**DOI:** 10.1007/s00393-022-01256-8

**Published:** 2022-08-31

**Authors:** Burkhard Möller

**Affiliations:** Universitätsklinik für Rheumatologie und Immunologie, Inselspital – Universitätsklinik Bern, Freiburgstr. 18P, 3010 Bern, Schweiz

**Keywords:** Bildgebung, Fallbericht, Chronische Rückenerkrankungen, Nichtsteroidale Antirheumatika, Biologische Therapieoptionen, Imaging, Case report, Chronic back diseases, Nonsteroidal anti-inflammatory drugs, Biological treatment options

## Abstract

**Hintergrund:**

Die axiale Spondyloarthritis (axSpA) ist unter den chronischen Rückenerkrankungen wohl die Entität mit dem größten Spektrum spezifischer antientzündlicher Behandlungsoptionen. Sie wird aber erst nach Rückenmark- oder Cauda-equina-Kompression, Knochenmetastasen, epiduralem Abszess oder Osteomyelitis der Wirbelkörper, Radikulopathie oder engem Spinalkanal nur als eine ferner zu berücksichtigende Ätiologie von Rückenschmerzen in den allgemeinmedizinisch orientierten Praxishilfen erwähnt. Es besteht wegen einer vergleichsweise niedrigen Prävalenz und erst später zu erwartender Folgen die tendenzielle Gefahr, dass die axSpA als eine Entität für Spezialisten vernachlässigt wird.

**Ergebnisse:**

Diese Arbeit rekapituliert die Empfehlungen der internationalen Gesellschaft für das Assessment der ankylosierenden Spondylitis (ASAS). Dieser Review weist auf die praktischen Aspekte der detaillierten Evaluation bisheriger Therapieversuche mit nichtsteroidalen Antirheumatika (NSAR) bei Rückenschmerzen hin. Von besonderem Interesse können dabei unerwünschte Effekte auf eine Symptomatik des unteren Intestinaltraktes sein. Die geschlechtsspezifischen Unterschiede im Ansprechen auf eine TNF(Tumor-Nekrose-Faktor)-Inhibitor-Therapie bei axSpA finden Erwähnung, ebenso wie weitere Aspekte der biologischen Therapieoptionen bei axSpA anhand eines Falles von anhaltender Remission einer HLA-B27 und Magnetresonanztomographie(MRT)-positiven axSpA nach Anti-IL(Interleukin)-17-Behandlung und dem mehrjährigen guten Ansprechen auf eine IL-12/23-Inhibitor-Therapie bei axialer Psoriasisarthritis besprochen werden. Ferner wird die Literatur im Hinblick auf Uveitis, Karditis und Amyloidose im Kontext der axSpA diskutiert.

**Schlussfolgerung:**

Die rechtzeitige Erkennung im allgemeinmedizinischen Kontext und die spezifische Berücksichtigung zahlreicher prädiktiver Faktoren spielen bei der personalisierten Behandlung der axSpA eine weiter zunehmende Rolle.

## Abstract (en)

**Background:**

Among chronic back diseases, axial spondylarthritis (axSpA) is the entity with the largest spectrum of specific anti-inflammatory treatment modalities; however, from a general medical perspective axSpA is only ranked as another etiology of back pain to be considered after spinal cord or cauda equina compression, bone metastases, epidural abscess or osteomyelitis of the vertebrae, radiculopathy or spinal stenosis. Due to its relatively low prevalence and mostly later occurring sequelae, there is a danger that axSpA will be a relatively neglected entity for specialists.

**Results:**

This article recapitulates the recommendations of the Assessment of Ankylosing Spondyloarthritis International Society (ASAS). This review addresses the practical aspects of the detailed evaluation of treatment attempts carried out so far with nonsteroidal anti-inflammatory drugs (NSAID) for back pain. Undesired effects on the symptoms of the lower intestinal tract could be of particular interest here. The sex-specific differences in the response to treatment with tumor necrosis factor (TNF) inhibitors in axSpA are mentioned. Further aspects of treatment options with biologics in axSpA are discussed based on a case of maintained remission after a course of interleukin (IL) 17 inhibitors in undifferentiated, HLA-B27 and magnetic resonance imaging (MRI) positive axSpA and long-standing good treatment response to IL-12/23 inhibitor treatment in axial psoriatic arthritis. Furthermore, the literature is discussed with respect to uveitis, carditis and amyloidosis in the context of axSpA.

**Conclusion:**

The early diagnosis in the general medical context and the specific consideration of numerous predictive factors play an increasingly more important role in the personalized treatment of axSpA.

Erst vor Kurzem ist von Kiefer et al. eine hervorragende Übersichtsarbeit zum Management der axialen Spondyloarthritis auf Basis der für Deutschland formulierten S3-Richtlinie in der *Zeitschrift für Rheumatologie* erschienen [10], in der nahezu alle aus unserer heutigen Sicht wichtigen Themen zu diesem Krankheitsbild aufgeführt werden. Was gibt es also schon wieder Neues zum Thema zu berichten, nachdem die Herausforderungen der sog. Frühdiagnose, das Monitoring der Erkrankung sowie Therapieziele und noch einmal separat das Thema Patientenschulung beleuchtet wurden?

Nun, die *Zeitschrift für Rheumatologie* ist nicht nur das Fachorgan der Deutschen Gesellschaft für Rheumatologie (DGRh), sondern auch der Österreichischen Gesellschaft für Rheumatologie und Rehabilitation (ÖGR) und der u. a. auch deutschsprachigen schweizerischen Fachgesellschaften für Rheumatologie (SGR [Schweizerische Gesellschaft für Rheumatologie]/SSR [Societé Suisse de Rhumatologie]). So erlaube ich mir, die Leserschaft über die Landesgrenzen hinweg zu einem vergleichenden Blick zur Versorgung der axialen Spondyloarthritis (axSpA) in verschiedene Versorgungssysteme einzuladen. Was passiert etwa, wenn Hausärzte oder Rheumatologen nicht sehr streng mit einem Budget gedeckelt sind? Oder was passiert hinsichtlich früher Diagnose und Biologikatherapie, wenn anstelle der in Deutschland üblicherweise mit Rückenschmerzen konfrontierten Orthopäden in der Schweiz Rheumatologen die primären Ansprechpartner für die Diagnostik und die nichtoperative Behandlung der chronischen Rückenschmerzen sind?

## Das Problem mit der Prävalenz, der Bildgebung, den roten und den gelben Flaggen

In der Primärdiagnostik von akuten Rückenschmerzen dürfte man sich zunächst am Vorliegen von Warnhinweisen für akute und äußerst schwerwiegende Probleme wie Rückenmark- oder Cauda-equina-Kompression, Knochenmetastasen, epiduralem Abszess oder Osteomyelitis der Wirbelkörper und auch nach einer Radikulopathie oder engem Spinalkanal orientieren. Die Studierenden der Medizin lernen diese Red-flag-Warnhinweise für umgehend zu behandelnde Ursachen, oft schon aber mit etwas weniger Inbrunst die „yellow flags“, worunter die meist psychosozial zu allozierenden Faktoren der Chronifizierung von Rückenschmerzen subsumiert werden.

Ein wesentliches Problem bei der zeitgerechten Diagnose der axSpA mag u. a. in dem Umstand begründet sein, dass der typische entzündliche Kreuzschmerz auch nicht bei den „yellow flags“ gelistet ist. Die Symptome des sog. entzündlichen Kreuzschmerzes tauchen in einer viel zitierten Liste unbedingt zu erfragender Symptome bei Rückenschmerzen in einer 10-min-Sprechstunde nicht auf [22]! Solange dieses der Fall bleibt, dürfte der entzündliche Rückenschmerz in der Konsultation lange unentdeckt bleiben, und es ist es somit auch nicht überraschend, dass die Zeitspanne bis zum Start der ersten axSpA-spezifischen medikamentösen Therapie in Europa im Mittel 6 Jahre dauert und diese Zeitspanne auch in der Schweiz nicht kürzer ist [4, 26].

Ist die entzündliche Rückenschmerzsymptomatik, wie so oft in der Praxis zu beobachten, nicht kontinuierlich vorhanden, so könnte sie als wiederkehrend akut auftretend interpretiert werden. Solange es zudem dank des typischen, guten Ansprechens auf nichtsteroidale Antirheumatika (NSAR) gelingt, die Symptomatik zu kontrollieren, würde der Sakroiliitis-bedingte Rückenschmerz auch nicht als praktisch relevantes Problem wahrgenommen werden. Bei Interpretation des Rückenschmerzes als wiederkehrend akut und weniger als 6 Wochen dauernd dürfte man es möglicherweise sogar als eine weise Entscheidung empfinden [26], dass man jahrelang auf eine entsprechende Bildgebung verzichtet. Die Diagnose wird nicht gestellt.

## Warum nur so spät?

Die Zeitspanne bis zur Verordnung eines „biological disease modifying antirheumatic drug“ (bDMARD) ist auch in der Schweiz sehr lang [4], wozu in einem Versorgungssystem ohne restriktive Vorgaben bezüglich Überweisung zum Facharzt, Durchführung einer Magnetresonanztomographie (MRT) oder zur Labordiagnostik andere als diese genannten Umstände angeführt werden müssen. Der Zugang zur MRT ist nicht reguliert, bezieht aber des Öfteren nicht die Kreuzbein-Darmbein-Gelenke mit ein. Ferner gibt es nach formlosem Antrag einer sog. Kostengutsprache kaum administrative Hürden für den Start einer In-label-Biologikatherapie zu überwinden. Insofern darf man annehmen, dass der im Mittel späte Beginn mit axSpA-spezifischen Therapeutika in etwa dem Verlauf der Symptomatik des Rückenschmerzes und der Wahrnehmung des Problems vonseiten der Ärzte und Patienten entspricht.

## Spezifische Anamnese des entzündlichen Rückenschmerzes

Die ASAS schlägt für die Diagnose der axialen Spondyloarthritis die Erhebung der folgenden 5 Kriterien vor: Rückenschmerzen länger als 3 Monate (1) mit schleichendem Beginn (2) vor dem 40. Lebensjahr (3), nächtliche Schmerzen mit Besserung bei Bewegung (4), aber nicht in Ruhe (5) [21]. Dieser Fragenkatalog fokussiert somit v. a. auf der Erfassung der axSpA-typischen Beschwerden bei der frühen ankylosierenden Spondylitis (AS) junger Männer. Nun startet die Spondylitis-Symptomatik bei Frauen nicht selten später als bei Männern. Auf die letzten Fälle meiner eigenen Sprechstunde bezogen, lohnte es sich deshalb, v. a. die Familienanamnese und die Fragen nach der NSAR-Verträglichkeit zu erheben. Insbesondere die Frage nach einer Diarrhö, die sich als Ausdruck einer Enteritis oder Kolitis durch den NSAR-Einsatz verstärkte, erleichterte, mit möglicherweise erheblichen Komplikationen verbundene ungünstige Entscheidungen bei der Differenzialindikation von Biologika zu vermeiden. Ferner empfiehlt es sich natürlich, nach schmerzhaften Augenrötungen, nach Knöchel‑, Knie- und Fersenschmerzen in der Vergangenheit des Patienten oder in der Familie zu fragen, um nur die für die Entdeckung einer Spondyloarthritis (SpA) aussichtsreichsten Konstellationen zu nennen.

## Klinische Untersuchung

Die klinische Untersuchung des Achsenskeletts beruht, abgesehen von der Beurteilung der Beweglichkeit, im Wesentlichen auf der Schmerzprovokation durch manuelle Manöver. In Ergänzung kann auch die gerne von Schmerzmedizinern angewendete Linderung durch Injektion von Lokalanästhetika zur approximativen Einordnung der Schmerzursache verwendet werden [17]. Weder das Handbuch der Assessment of Spondyloarthritis International Society (ASAS) noch deren sehr empfehlenswerte Lehrbildsammlung enthalten aber derzeit diesbezügliche Vorschläge. In einer aktuell durchgeführten Literaturrecherche führte die Suche nach Arbeiten mit den Titel- oder Abstract-Suchbegriffen „sacroiliitis“ & „physical“ & „examination“ zu insgesamt nur 75 Arbeiten. In 3 aus den letzten 2 Jahren stammenden Arbeiten wurden darin 5 der allein mir bekannten 8 Varianten von Stresstests für die Iliosakralgelenke evaluiert, aber keiner dieser ISG(Iliosakralgelenk)-Provokationstests zeigte eine für die Praxis ausreichende Sensitivität und Spezifität für die Diagnose einer Sakroiliitis oder axSpA [17, 24].

## Bildgebung

**Figure 1:**
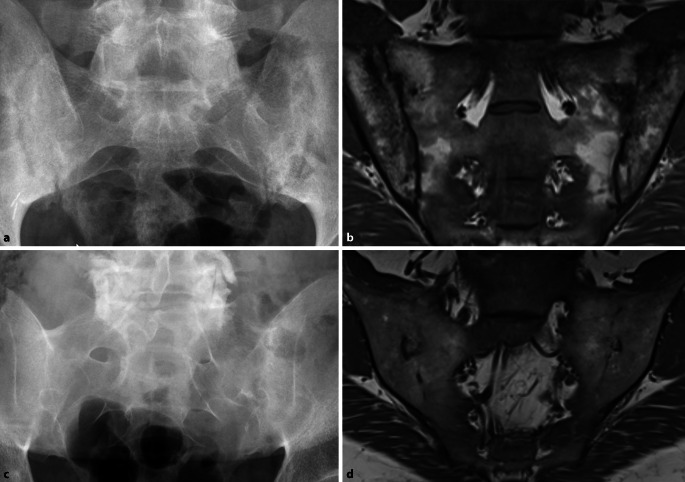

**Figure 2:**
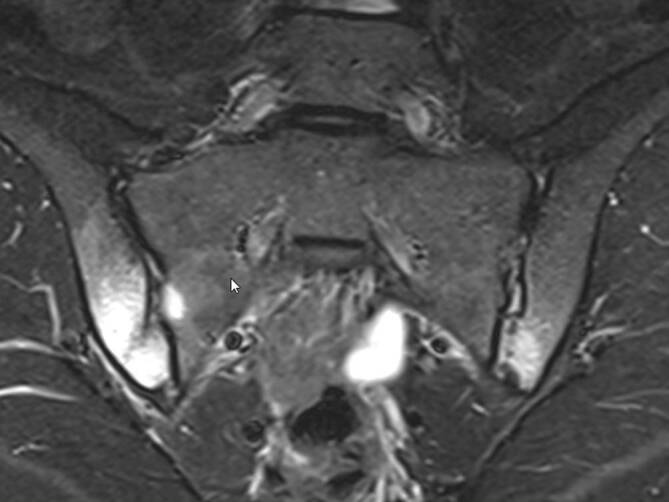

Da die irreversiblen Einschränkungen der Wirbelsäulenbeweglichkeit oder der Thoraxexkursion fortgeschrittene Stadien der AS darstellen, ist der konventionell-radiologische Nachweis einer zumindest beidseitigen Grad-II-Sakroiliitis oder einer einseitigen Grad-III-Sakroiliitis (Abb. 1) ein entscheidendes Kriterium für die Diagnose einer AS [11]. Die aufgrund der konventionell-radiologisch erkennbaren Pathologien postulierte Prädominanz dieses Krankheitsbildes bei männlichem Geschlecht, jungem Alter und HLA-B27-Positivität wurde nach der späteren Einführung der Magnetresonanztomographie (MRT) relativiert, indem die MR-tomographisch fassbare axSpA bei Frauen wie Männern gleichermaßen häufig vorkommt und zudem weder auf das Alter unter 40 oder 45 Jahren noch auf HLA-B27-Träger beschränkt ist [19]. Die MRT erlaubt den Nachweis der entzündlichen Aktivität der Sakroiliitis [14] wie auch der Spondylitis [3] anhand des in T2- oder den sog. STIR(„short tau inversion recovery“)-Sequenzen nachweisbaren Knochenmarködems (Abb. 2). Mit der Zeit kommt es neben Erosionen, definiert als Verlust der Kontinuität der subchondralen Kompakta und verminderte Signalintensität, auch in T1-gewichteten MRT-Sequenzen (Abb. 1d) zu diagnostisch verwertbaren Pathologien [15]. Später werden diese Erosionen dann durch fettreiches, in T1 hyperintenses Ersatzgewebe aufgefüllt, das auch als Backfill bezeichnet wird. Das gelenknahe Knochenmark wird zudem durch in T1 homogen hyperintenses, scharf begrenztes metaplastisches Fettgewebe ersetzt, während es aber auch zur vollständigen Ankylose der Kreuzbein-Darmbein-Gelenke ohne jede Verfettungstendenz kommen kann. Gemäß Spondyloarthritis Research Consortium of Canada(SPARCC)-Score gibt es in einem Quadranten der ISG entweder Erosion oder Backfill, aber nicht beides gleichzeitig [15].

Der Fokus der Untersuchung liegt auf den Kreuzbein-Darmbein-Gelenken

In Analogie zur konventionellen Röntgendiagnostik und den modifizierten New-York-Kriterien liegt auch bei der MRT der Fokus auf der Untersuchung der Kreuzbein-Darmbein-Gelenke. Aktuelle MRT-Daten der „SpondyloArthritis-Caught-Early“(SPACE)-Kohorte zeigen, dass die klassischen vertebralen Zeichen der Spondylitis isoliert oder zumindest vor jeglichem ISG-Befund insbesondere an der thorakalen Wirbelsäule nachgewiesen werden können [13]. Aufgrund dieser Daten muss bei der Abklärung von Verdachtsfällen einer SpA eine MRT des gesamten Achsenskeletts oder zumindest der symptomatischen Abschnitte empfohlen werden.

## Medikamentöse Therapie

Wir kennen weder für die AS noch für die axSpA kurative Therapieansätze, obwohl dem Autor über mehr als 2 Jahre anhaltende komplette Remissionen, etwa der Spondyloarthritis einer 32-jährigen HLA-B27-positiven Patientin mit MR-tomographisch gesicherter Sakroiliitis, voluminöser und im Gelenkpunktat sehr zellreicher peripherer Arthritis, Daktylitis und klinisch diagnostizierter Synchondritis sternalis nach nur 1‑monatiger Anti-IL(Interleukin)-17-Antikörpertherapie, bekannt sind. Über etliche Monate anhaltende therapeutische Effekte insbesondere einer hoch dosierten Anti-IL-17-Therapie sind bereits seit Veröffentlichung der MEASURE-1-Studie beschrieben [26]. Derart rasche und gleichermaßen anhaltende Behandlungserfolge bleiben nach meiner Erfahrung die große Ausnahme. Die Regel ist viel eher der chronisch schleichende Krankheitsbeginn, die partielle Wirksamkeit von NSAR und somit die Frage des rechten Zeitpunktes einer sog. zielgerichteten, d. h. „targeted biological“ oder „synthetic“ DMARD-Therapie. Basierend auf den für jede der Substanzen durchgeführten Phase-III-Studien, sind in der EU und in der Schweiz für die Behandlung der AS alle TNF(Tumor-Nekrose-Faktor)-Inhibitoren inklusive deren Biosimilars, die IL-17-Inhibitoren Secukinumab und Ixekizumab und der Januskinaseinhibitor Upadacitinib zugelassen. Im Gegensatz zu der bei der axSpA für einige Biologika an eine „objektivierte“ Entzündung in der MRT oder im Labor geknüpften Zulassungssituation in Deutschland bzw. in der EU sind in der Schweiz ganz generell nicht alle Biologika, sondern lediglich Adalimumab, Certolizumab, Etanercept und Secukinumab für die Behandlung der nichtradiographischen axSpA zugelassen.

## Krankheitsprogression

Eine progrediente Ankylose betrifft nur den kleineren Anteil aller Patienten mit einer axSpA, und diese verläuft in der Regel äußerst langsam. Die Vermeidung struktureller Veränderungen verlangt deshalb lange Behandlungszeiten bzw. deren Nachweis in Studien Beobachtungszeiträume von zumindest 4 Jahren [8]. Die TNF-Blocker-Effekte zur Vermeidung einer Ankylose finden sich dabei v. a. bei Patienten, bei denen bereits Jahre früher auch ein am ASDAS (Ankylosing Spondylitis Disease Activity Score) gemessenes therapeutisches Ansprechen zu beobachten war [18]. Diese Beobachtungsdaten aus dem SCQM (Swiss Clinical Quality Management in Rheumatic Diseases) sprechen somit sehr für ein regelmäßiges ASDAS-Monitoring und suggerieren damit ferner die Möglichkeiten, mit einem derartigen „treat to target“ die progrediente Ankylose der AS vermeiden zu können.

## Extraspinale Manifestationen

**Figure 3:**
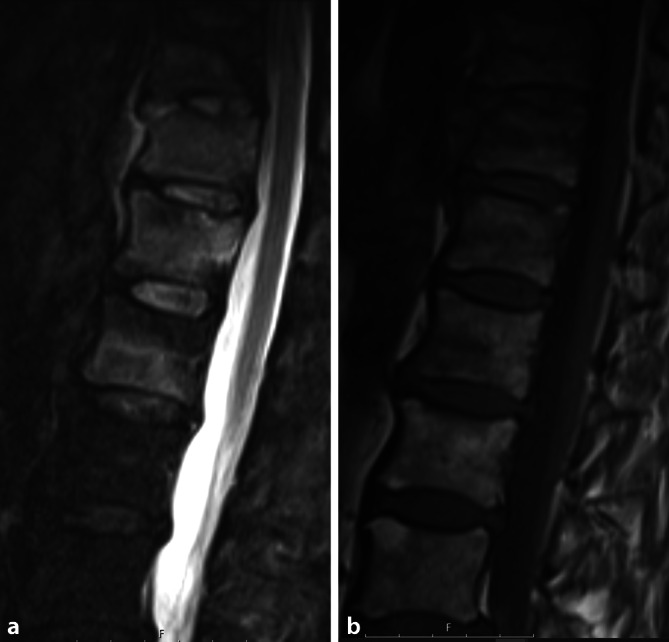

Sofern bei der axSpA auch eine Psoriasis vorliegt, so können im Zuständigkeitsbereich der Swissmedic alle in der Schweiz bei der Psoriasisarthritis (PsA) zugelassenen Medikamente im Sinne einer sog. „ax(axiale)PsA“ zum Einsatz kommen. Dieses schließt somit auch den Anti-IL-12/23-p40-Antikörper Ustekinumab sowie die p19-spezifischen Anti-IL-23-Antikörper Guselkumab und Risankizumab ein, die bei der klassischen axSpA nicht eingesetzt werden sollen. Wie bereits früher für Ustekinumab gezeigt, gibt es aber mittlerweile für Guselkumab wie auch in Post-hoc-Analysen der Discover-1- und -2-Studien Hinweise auf eine dem Placeboarm überlegene Wirksamkeit bei der axPsA [7, 9]. Die Abb. 3 zeigt den Fall einer bei Therapiebeginn 57-jährigen, HLA-B27-negativen Patienten mit Plaquepsoriasis, peripherer Arthritis, deutlich erhöhter alkalischer Phosphatase im Serum und einer Synchondritis sternalis, also einem sog. „anterior chest wall syndrome“, die bei fast symptomfreier Haut, Remission der peripheren Arthritis, deutlich gebesserten Rücken- und Thoraxschmerzen und anhaltend, aber nicht komplett normalisierter alkalischer Phosphatase mittlerweile 6 Jahre lang von einer Ustekinumab-Therapie profitieren konnte. Da es im Gegensatz zur axialen PsA keine positiven Studienresultate für IL-12/23- oder IL-23-Antikörperbehandlungen bei der axSpA oder AS gibt, ist die Identifikation einer Psoriasis nicht mehr nur von akademischem Interesse. Eine weitere zu bedenkende Komponente bei der Therapieauswahl der axSpA könnte in der Zukunft das Geschlecht des Patienten bzw. der Patientin darstellen, da aus bislang unerklärlichen Gründen, aber wiederholt im klinischen Alltag gezeigt, Frauen signifikant schlechter als Männer auf eine TNF-Inhibitor-Therapie ansprechen [5].

Frauen sprechen auf eine TNF-Inhibitor-Therapie signifikant schlechter an als Männer

Eine große Bedeutung für die Auswahl eines der verschiedenen bei der axSpA indizierten TNF-Inhibitoren oder eines IL-23- vs. eines IL-17-spezifischen Antikörpers hat der intestinale Befall [23]. Zudem sind für die Behandlung der Uveitis im Kontext einer axSpA gemäß Literatur nicht gleichermaßen alle TNF-Inhibitoren, sondern v. a. Adalimumab und Infliximab einzusetzen [1]. Die Erfahrungen mit den Anti-IL-17- und Anti-IL-12/23-Therapeutika sind bei der SpA-Uveitis überwiegend positiv [25], aber nicht nur rein zahlenmäßig geringer, sondern auch im Ergebnis tendenziell weniger günstig als mit TNFi (Tumor-Nekrose-Faktor-Inhibitor). Zudem sind auch Flares einer idiopathischen Uveitis unter Ustekinumab [6] wie auch im Vergleich zu TNFi höhere Uveitis-anterior-Raten bei Secukinumab beobachtet worden [12].

Die Amyloidose ist gemäß einer landesweit zwischen 1999 und 2015 in Spanien durchgeführten Hospitalisierungsstudie mit einer Prävalenz von im Mittel 0,7 % eine seltene Komplikation der axSpA, die zudem häufiger bei gleichzeitig vorliegender chronisch entzündlicher Darmerkrankung bzw. „inflammatory bowel disease“ (IBD) gefunden wurde, v. a. aber in der Gesamtgruppe von Jahr zu Jahr im Mittel um 4,6 % abgenommen hat [16]. Insgesamt aber hatte die Amyloidose in dieser Studie als Komplikation anderer Entitäten zugenommen, sodass es keinen offensichtlicheren Grund für den rückläufigen Trend der Amyloidose bei der axSpA als den Einsatz der Biologika gibt. Schließlich sei noch an die erhöhte Inzidenz einer Herzbeteiligung bei über 50-jährigen AS-Patienten erinnert, die etwa 5‑mal häufiger als bei altersentsprechenden Kontrollen mit einer Arthrose gefunden wird. Klinisch handelt es sich dabei aber um funktionell überwiegend irrelevante diastolische Störungen der linksventrikulären Funktion, während die früher klassische Aorteninsuffizienz oder auch die Mitralinsuffizienz zahlenmäßig kaum zu Buche schlägt [2, 20].

## Fazit für die Praxis

- Die Diagnostik von chronischen oder rezidivierenden Rückenschmerzen ist im Wesentlichen im Zuständigkeitsbereich der Hausärzte und Allgemeinmediziner, in Deutschland wohl auch der orthopädischen Chirurgie angesiedelt.
- Die medikamentöse Therapie der axialen Spondyloarthritis (axSpA) gehört hingegen in die Hände immunologisch spezialisierter Rheumatologen, die weiterhin die enge Zusammenarbeit mit den Primärversorgern für Rückenschmerzpatienten suchen müssen.
